# National Prescribing Trends and Cost Analysis of Antidepressants, Anxiolytics, and Hypnotics in England, 2010–2023

**DOI:** 10.1002/hup.70011

**Published:** 2025-08-18

**Authors:** Mike Stedman, Mark Davies, John Warner‐ Levy, Joseph Ingram, David Taylor, Adrian Heald

**Affiliations:** ^1^ Res Consortium Andover UK; ^2^ University of Manchester Manchester UK; ^3^ Greater Manchester Mental Health Manchester UK; ^4^ Institute of Psychiatry London UK; ^5^ Department of Endocrinology Salford Royal Hospital Salford UK

**Keywords:** antidepressant, anxiolytic, general practice, hypnotic, prescribing, trends

## Abstract

**Introduction:**

We describe prescribing of anxiolytics/hypnotics/antidepressants at an all‐England level between 2010 and 2023, taking account of the influence of the COVID‐19 pandemic‐period.

**Methods:**

All Primary Care Prescribing data for England for anxiolytics/hypnotics/antidepressant agents taken as tablets or capsules from 1 January 2010 to 31 December 2023 were considered.

**Results:**

Antidepressants: the greatest increases were in sertraline prescriptions increasing from 2.9million 2010 by 685% to 23.0 million‐2023; duloxetine increased by 545% to 3.9million prescriptions; mirtazapine by 269% to 12.4million prescriptions. Regarding cost, overall average cost fell 2010‐2023 by 54% from £4.86(Euros 5.68) to £2.25(Euros 2.63) per prescription. Anxiolytics: the number of prescriptions for anxiolytics fell 6.5–4.8 m (−27%) overall, with all anxiolytics falling except buspirone. The greatest period of fall was seen between 2018 and 2023. Lorazepam/diazepam/chlordiazepoxide were down 23%,16%, 88% respectively. Average cost of prescriptions fell by 13% to £3.20(Euros 3.74). Hypnotics: the number of prescriptions for hypnotics fell 41% from 9.8million to 5.8million in 2023. All prescriptions fell in number. Annual trends showed no deviations for the COVID‐19 core pandemic (2020/2021) years.

**Conclusion:**

Overall trends demonstrate a more than doubling of antidepressant prescribing over the period 2010–2023, with similar volume reduction in anxiolytics/hypnotics over the same period. Such real world data facilitate the development of policy influencing insights for psychotropic prescribing management now and in coming years.

## 1 Introduction

Prescribing trends in common psychotropic medications reflect changes in therapeutic options and public perception of mental illness (Sanborn et al. 2023). Robust evidence about trends in psychotropic prescribing should be derived using data with sufficiently long follow‐up periods to ensure that any changes observed are not due to systematic bias.

Antidepressant prescribing in England has generally shown an upward trend over the last 30 years, with an increase in both the number of items prescribed and the number of patients receiving them (Bogowicz et al. 2021). In contrast, a declining trend in antipsychotic prescribing has been observed internationally and is attributed to the release of guidelines, safety warnings and tightened prescribing restrictions (Harrison et al. 2020).

The early months of the COVID‐19 pandemic saw a significant increase in telehealth services especially for mental health services (Zhu et al. 2022). What has remained less clear is how prescribing of specific psychotropic classes may have changed as part of this shift in service delivery.

In a recently published network metaanalysis (Luo et al. 2024), looking at prescribing in patients with depressive and anxiety disorders before and during the COVID‐19 pandemic across diverse study populations and health‐care settings, the authors found an overall immediate increase in psychotropic drug prescribing across databases in the early stages of the pandemic with continued longer term elevated rates of anxiolytic prescribing in France, the United Kingdom (UK) South Korea and the United States. However the average monthly rates of patients prescribed psychotropic drugs differed substantially between some of the national databases.

We previously described an analysis of prescribing data for all of England in the time before, during and after the main impact period of the COVID‐19 pandemic (Waheed et al. 2023). We reported a slight fall in the main antidepressant agents prescribed, while for anxiolytics, the trends over time were in the opposite direction.

Here we describe an extension of our previous work using all England general practice prescribing data, to investigate how the prescribing of antidepressants and anxiolytics/hypnotics at an all‐England level changed between 2010 and 2023, taking account of the influence of the COVID‐19 pandemic period.

## 2 Methods

The annual primary care prescribing cost analysis (PCA) data for each year in England for 2010 to 2018 were downloaded from NHS Digital (https://digital.nhs.uk/data‐and‐information/publications/statistical/prescription‐cost‐analysis) and from 2019 to 2023 from National Health Service Business Services Authority NHSBSA for all medication prescribing in general practices in that year. This annual data included for each BNF code the medication agent, tablet size in milligrammes, the total number of prescriptions and quantity of tablets and actual costs. Data for BNF Sections 4.1 and 4.3 covering anxiolytics, hypnotics and anti‐depressant agents were then extracted. The dose level of each code item for those taken as tablets or capsules was linked to the given mcg quantity for that BNF code to establish the total mcg for each code.

In order to contextualise the levels of these change between 2010 and 2023 we reported the growth in total population (ONS Census 2021) and rise in consumer price index of CPI as published by the ONS (ONS Consumer Price Index). There have also been other changes in social deprivation, ethnicity and other non‐medical demographic factors during this period that would have moderated prescribing. However these have not been investigated or reported here.

This data is a consolidation of the complete total national prescribing of all general practices in England. The numbers of patients receiving the medication is not given, and so the average dose and costs reflected the average dose and costs over all prescriptions of a particular medication in that year, not that prescribed to the average patient. We looked at change in duration of prescriptions, availability of different dose levels and aggregation of more than one dose size to one patient to prevent simple patient level comparison. It was understood that within these annual figures were included many changes in patient level use and applications. However the overall trends were of interest in themselves.

Cost reporting is not a simple issue, as different medications are charged and reimbursed at different rates. We have used the Net Ingredient Cost (NIC) as reported in the PCA which is the basic price of the medication and the quantity prescribed. It does not include other fees incurred by dispensing contractors, such as controlled drug fees or the single activity fee. The basic price is determined by the Drug Tariff or by the manufacturer, wholesaler, or supplier of the product.

For each overall medication in each year we considered.

- Total number of prescriptions
- Average cost/prescription
- Average dose/prescription

In order to establish the relative percentage (%) change, the value in each year was divided by the original value in 2010 as equal to 100%.

This analysis was conducted by aggregating and filtering the large annual csv files using Delimit software and then analysing the consolidated 2010‐23 data using Excel Power Pivot.

### 2.1 Ethical Considerations

Ethical approval was not considered to be needed as we analysed publicly aggregated general practice level data.

## 3 Results

Evaluation of these trends should be considered against the 9.5% growth in total population given by the ONS as 52.6 million in 2010 to 57.7million in 2023 and rise in consumer price index of CPI 146% in 2023 compared to 2010 (Table 1).

**TABLE 1:** Total prescribing between 2010 and 2023 in practices in England Source data https://www.nhsbsa.nhs.uk/statistical-collections/prescription-cost-analysis-england/prescription-cost-analysis-england-202324.

BNF chapter: 4		
BNF sect	Medication	Prescriptions
4.3	Citalopram Hydrobromide	193,918,566
	Amitriptyline	177,492,806
	Sertraline	174,364,670
	Mirtazapine	110,202,858
	Fluoxetine	89,517,371
	Venlafaxine	56,945,697
	Duloxetine	28,775,305
	Paroxetine	19,605,595
	Escitalopram	16,959,321
	Trazodone	14,795,408
	Dosulepin	12,901,952
	Nortriptyline	8,350,498
	Clomipramine	4,040,108
	Lofepramine	3,145,680
	Imipramine	2,178,871
	Flupentixol	2,081,794
	Vortioxetine	1,343,245
	Trimipramine Maleate	852,126
	Doxepin	472,688
	Reboxetine	441,822
	Fluvoxamine Maleate	355,275
	Agomelatine	313,935
	Moclobemide	233,082
	Phenelzine Sulphate	192992
	Tranylcypromine Sulphate	105413
	Mianserin	60,130
	Tryptophan	54,588
	Isocarboxazid	24,234
	Nefazodone	2,440
	Oxitriptan	597
	Amoxapine	47
	Maprotiline	19
	Other Antidepressant Preparations	20
	Protriptyline	6
	Total	919,729,159

**Delivery: Cap/Tab**		
**BNF Sect**	**BNF medication name**	**Prescriptions**
4.1	Zopiclone	74,134,560
	Diazepam	69,209,366
	Temazepam	19,427,423
	Lorazepam	10,153,050
	Zolpidem Tartrate	9,588,606
	Nitrazepam	8,356,975
	Buspirone	2,424,937
	Oxazepam	1,448,078
	Chlordiazepoxide	1,338,139
	Loprazolam Mesilate	605,428
	Lormetazepam	390,973
	Clomethiazole	372,518
	Cloral Betaine	108,811
	Other Hypnotic Preps	95,108
	Meprobamate	32,394
	Secobarbital Sodium	30,482
	Amobarbital Sodium	25,782
	Zaleplon	19,823
	Butobarbital	13,192
	Daridorexant	172
	Alprazolam	163
	Bromazepam	67
	Flurazepam	35
	Amobarbital	28
	Total	197,776,110

The change in annual number of prescriptions, average dose per prescription and cost per prescription are given in Supporting Information S1: Tables 1 and 2 for all the agents described.

### 3.1 Antidepressants

**FIGURE 1:**
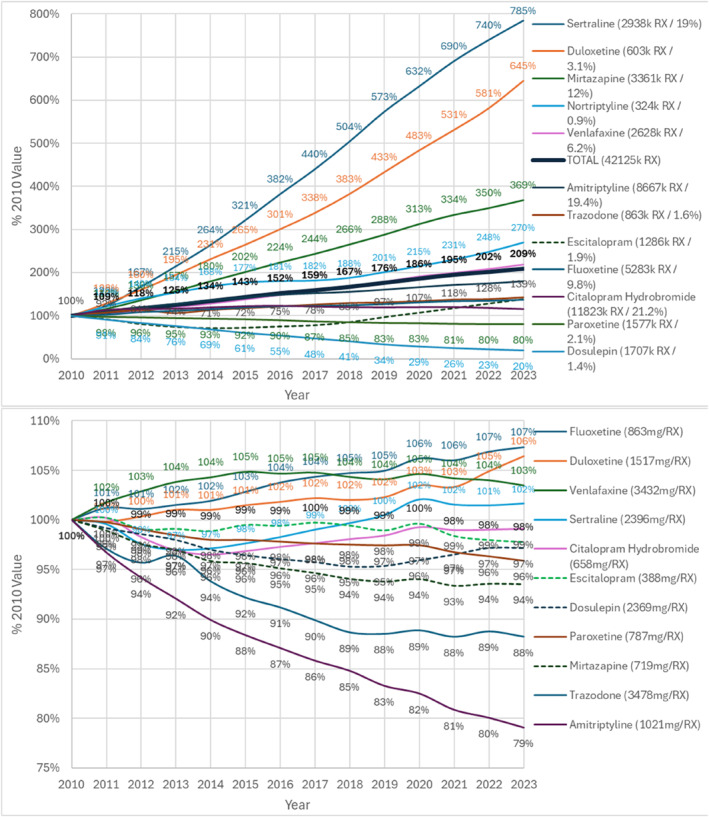
Antidepressant development by year as % 2010 value = 100% (A) Total prescriptions legend = (2010 value/% of 2010 total) (B) Average dose mg/prescription legend = (2010 value).

Total 42 million prescriptions in 2010 increasing by109% to 88 million in 2023. The greatest increases were in sertraline prescriptions increasing from 2.9 million in 2010 by 685% to 23.0 million, in duloxetine, increasing by 545% to 3.9 million prescriptions and in mirtazapine, increasing 269% to 12.4 million prescriptions (Figure 1a). Paroxetine prescriptions fell by 20% to 1.26 million.

The largest fall in dose (Figure 1b) was for amitriptyline by 21% from 1021–807 mg/prescription and trazodone by 12% from 3478 to 3068 mg/prescription. While the average dose of fluoxetine increased by 7% from 863 to 926 mg/prescription and duloxetine by 6% from 1517 to 1615 mg/prescription. The average of the % change in mg/prescription over all agents over the period weighted by number of prescriptions in 2023 was a fall of 4%.

Regarding cost, overall average cost fell between 2010 and 2023 by 54% from £4.86 (Euros 5.68) to £2.25 (Euros 2.63) per prescription. The largest reductions were for escitalopram by 91% from £19.30 (Euros 22.58) to £1.66 per prescription (Euros 1.94) and duloxetine by 86% from £28.00 (Euros 32.76) to £3.79 (Euros 4.43) per prescription. Dosulepin increased by 437% from £2.38 (Euros 2.78) to £13.31 (Euros 15.57) per prescription and clomipramine increased by 92% from £7.29 (Euros 8.53) to £14.01 (Euros 16.39) per prescription. The overall average % change in costs between 2010 and 2023 weighted by number of prescriptions in 2023 was a reduction of 36%.

### 3.2 Anxiolytics

**FIGURE 2:**
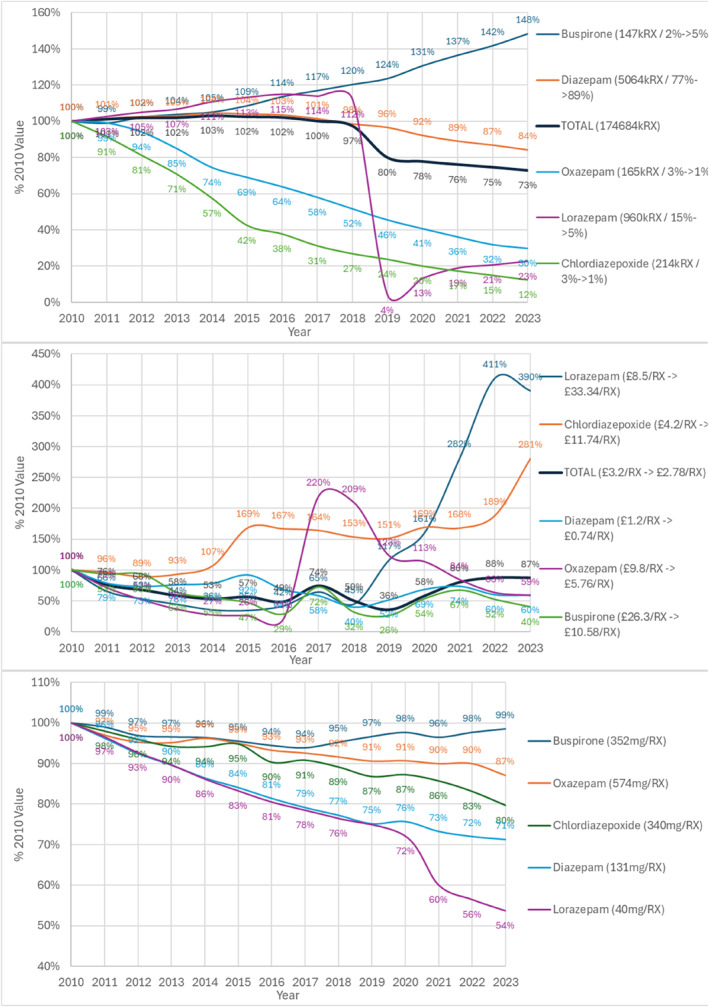
Anxiolytics development by year as % 2010 value = 100% (A) Total prescriptions legend = (2010 value/% 2010 total ‐> % 2023 total) (B) Average cost/prescription legend = (2010 value/2023 value) (C) Average dose mg/prescription legend = (2010 value).

Total 6.5 million prescriptions in 2010 fell 27% to 4.8 million in 2023 (Figure 2a). All agents fell between 2010 and 2023, except buspirone. The main period of fall was between 2018 and 2023. Lorazepam prescriptions fell precipitately in 2020 and did not return to their previous level (23% of 2010 level in 2023 at 216,000 prescriptions). Diazepam reduced by 16% to 4.3 million prescriptions and buspirone increased by 48% 147,000 to 217,000 prescriptions in 2023. Chlordiazepoxide decreased by 88% from 213.000 to 26,000 prescriptions in 2023.

Regarding cost, overall average cost fell by 13% to £2.78 (Euros 3.30) per prescription (Figure 2b), with a fall for buspirone of 60% from £26.30 (Euros 30.77) to £10.60 (Euros 12.40)) per prescription while lorazepam increased from 2019 onwards by 290% from £8.50 (Euros 9.95) to £33.30 (Euros 38.96) per prescription. Chlordiazepoxide increased by 181% to £11.74 (Euros 13.73) per prescription.

The largest fall in dose was for lorazepam by 46% from 40 to 21 mg/prescription, and diazepam by 29% from 131 to 94 mg/prescription (Figure 2c). The average of the % change in mg/prescription over all agents over the period weighted by number of prescriptions in 2023 was a fall of 28%.

### 3.3 Hypnotics

**FIGURE 3:**
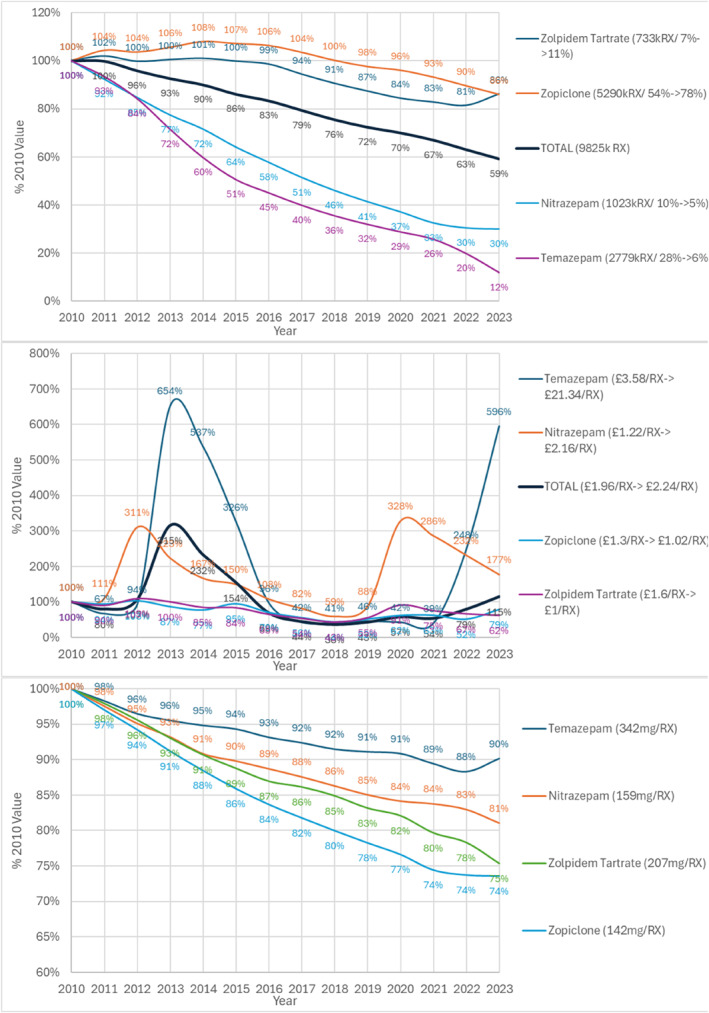
Hypnotic development by year as % 2010 value = 100% (A) Total prescriptions legend = (2010 value/% 2010 total ‐> % 2023 total) (B) Average cost/prescription legend = (2010 value/2023 value) (C) Average dose mg/prescription legend = (2010 value).

Total 9.8 million prescriptions in 2010 fell 41% to 5.8 million in 2023 (Figure 3a). All agents fell with temazepam falling the most from 2.8 million by 88%–333,000 in 2023. Zopiclone fell by 14% to 4.5 million, zolpidem fell by 14%–630,000 and nitrazepam by 70% between 2010 and 2023 to 306,000 prescriptions.

Regarding cost, overall average increased slightly by 14% to £2.24 (Euros 2.67) per prescription (Figure 2b), The cost of zolpidem decreased by 38% to £1.60 (Euros 1.87) per prescription and of zopiclone by 21% to £1.30 (Euros 1.52) per prescription (Figure 3b). The cost of temazepam (volume reduced by 2023 to 12% of 2010) increased by 496% from £3.58 (Euros 4.19) to £21.34 (Euros 24.97) per item.

The largest fall in dose was for zopiclone by 26% from 142 to 102 mg/prescription, and zolpidem by 25% from 207 to 156 mg/prescription Figure 3c. Average of the % change in mg/prescription over all agents over the period weighted by number of prescriptions in 2023 was a fall of 25%.

Annual trends showed no deviations for the years containing COVID‐19 core pandemic (2020/2021) and continuing trend in the years after the pandemic.

## 4 Discussion

Using all England general practice prescribing data from 2010 to 2023, we found that while prescribing of antidepressants has been growing at 5.4% per annum (pa), anxiolytics have been falling at 2.2%pa and hypnotics at 3.6% pa. Overall antidepressant prescribing increased by 109% between 2010 and 2023 and anxiolytic prescribing fell by 27% and hypnotic prescribing by 41%.

The downward trend in anxiolytic prescribing parallels trends seen in other studies (). We speculate that one factor in our results, may be initiatives in the United Kingdom NHS context to encourage alternative therapeutic strategies. We also found that the mg dose in each prescription for anxiolytics/hypnotics has also been falling annually by 1.6%pa. Overall, this has greatly reduced the amount prescribed for the two therapies during the 14 years studied.

Bogowicz et al. in 2023 reported that the number of antidepressant items prescribed per year in England more than tripled over 2 decades with similar findings in an earlier general practice database data study, which included data for 1995–2011 (Mars et al. 2017). Similar trends have been noticed elsewhere in the world (Mojtabai and Olfson 2014; Huijbregts et al. 2017). A contributory factor is likely to be longer‐term prescribing which may partly be due to difficulties stopping antidepressants and the perceived safety record of selective serotonin reuptake inhibitors (SSRIs) for long‐term treatment when compared with the older antidepressants. Longer‐term prescribing is also standard practice for prevention of relapse in recurrent depression (Geddes et al. 2003).

Evidence was also reported by Bogowicz et al. (2021) of significant variation in prescribing at practice level, by class, and for specific drugs such as dosulepin. It is likely that some of this variation may be warranted, accounted for by case‐mix and patient preference with some accounted for by individual prescriber preference.

The rise in antidepressant prescriptions may relate to changes in the wider society and the way these impact on psychological health. Notably recently published data from the NHS Business Services Authority (NHSBSA medicines used in mental health, 2024) indicated that an estimated 89 million antidepressant drug items were prescribed, an increase of 3.3% in just 1 year. The antidepressants also had the largest number of patients, an increase of 2.1%–8.7 million compared to 2022//2023.

People prescribed antidepressants are more likely to stop treatment than to continue for 6 months (Sawada et al. 2009) and this is often done without the awareness of the prescriber). Patient‐determined antidepressant cessation is likely to be abrupt and to occur before the end of the recommended treatment period (Woodward et al. 2016). Both of these factors predict depressive relapse and subsequent restart of antidepressants.

Our findings in relation to decrease in anxiolytic prescribing contrast with those of Archer et al. 2022, who described an increase in anxiolytic prescribing between 2003 and 2018. However much of the decrease in anxiolytic prescribing that we describe as between the years 2018 and 2023 which were not covered by the Archer analysis. The precipitate fall in lorazepam prescribing in 2020 relates to national and international manufacturer supply issues at the time (Pfizer Information to Healthcare Professionals).

The decrease in anxiolytic prescribing in England may well reflect the NICE recommendations regarding treatment of anxiety (www.nice.org.uk/guidance/cg113) and the availability of non‐pharmacological interventions such as those delivered by Talking Therapies and similar services (www.nhs.uk/mental‐health/talking‐therapies).

This finding is contrary that of Luo et al. in 2024, who described for some regions of the world a continued increase in anxiolytic prescribing as the COVID‐19 pandemic progressed, including in the UK. However the data from the OK that the authors accessed for the Luo et al. analysis was from the IQVIA database with covers 6 million people not the whole of England, as was the case for our study.

Psychotropic medication and psychotherapy are both effective in treating most mental health conditions. There is some evidence that combined treatments may be more effective than each of these treatments alone (NIMH 2023 National Institute of Mental Health). The literature also shows that receipt of psychotherapy enhances adherence to medication (NIMH 2023). Nevertheless many patients with mental health conditions receive psychotropic medication without any psychotherapy (Olfson and Marcus 2010).

Regarding hypnotic prescribing our findings are similar to those from an Australian cohort study looking at the period 2011–2018 (Begum et al. 2021). The authors reported an overall decreased in benzodiazepine prescriptions between 2011 and 2018. Similar findings were reported by Hughes et al. 2016, who described a reduction in benzodiazepine prescriptions in primary care alongside increases in nonbenzodiazepine and melatonin prescribing.

Regarding prescribing cost, the cost of 3 psychotropic agents increased factorially between 2010 and 2023, lorazepam by 3.90‐fold, chlordiazepoxide by 2.81‐fold, temazepam by 5.96‐fold and dosulepin 5.37‐fold (Table 2b, Figures 2b and 3b and Supporting Information S1: Tables 1 and 2). This was in tandem with a marked reduction in prescribing of all these agents. With the exception of chlordiazepoxide these price per item increases have largely offset the decrease in volume over time with the total spend for lorazepam in 2023 £7.2million compared with 8.2 million in 2010; total spend for temazepam in 2023 £7.1million compared with £10.0 million in 2010; total spend for dosulepin in 2023 £4.6 million compared with £4.2 million in 2010.

**TABLE 2:** BNF 0401 Prescribing analysis by Medication Anxiolytics and Hypnotics taken as tablets/capsules in 2023 versus. 2010.

Year	Medication	Total				‘/prescription (Px)			Pxs % total		Compared to 2010						
		Prescriptions	Quantity	Cost	Mg	Mg	Cost/					Pxs	mg/Pxs			£/Px	
Anxiolytics																	
2010	Buspirone	146,842	7,995,304	£3,866,333	51,624,345	352		£26.33	2%								
	Chlordiazepoxide	213,761	9,915,081	£894,219	72,683,000	340		£4.18	3%								
	Diazepam	5,064,356	174,683,957	£6,278,497	665,747,739	131		£1.24	77%								
	Lorazepam	960,191	35,147,006	£8,206,890	38,121,580	40		£8.55	15%								
	Oxazepam	164,812	8,028,304	£1,615,597	94,590,700	574		£9.80	3%								
	Total	6,549,962	235,769,652	£20,861,536	922,767,364	141		£3.18									
2023	Buspirone	217,868	11,616,181	£2,304,989	75,464,838	346		£10.58	5%			148%	99%		40%		
	Chlordiazepoxide	26,165	1,016,398	£307,220	7,085,750	271		£11.74	1%			12%	80%		281%		
	Diazepam	4,256,005	111,390,242	£3,145,879	398,516,321	94		£0.74	89%			84%	71%		60%		
	Lorazepam	216,567	6,946,268	£7,219,982	4,616,791	21		£33.34	5%			23%	54%		390%		
	Oxazepam	49,102	2,095,900	£283,000	24,530,120	500		£5.76	1%			30%	87%		59%		
	Total	4,765,707	133,064,989	£13,261,069	510,213,819	107		£2.78				73%	76%		87%		
Hypnotics																	
2010	Nitrazepam	1,022,852	32,527,845	£1,246,514	162,639,225	159	£1.22		10%							
	Temazepam	2,778,874	76,531,914	£9,954,461	950,698,130	342	£3.58		28%							
	Zolpidem tartrate	733,016	19,539,712	£1,175,622	151,891,310	207	£1.60		7%							
	Zopiclone	5,289,867	125,202,630	£6,858,848	748,584,619	142	£1.30		54%							
	Total	9,824,609	253,802,101	£19,235,445	2,013,813,284	205	£1.96		100%							
2023	Nitrazepam	306,782	7,909,145	£662,551	39,545,725	129	£2.16		5%	30%			81%	177%		
	Temazepam	333,124	8,051,369	£7,110,066	102,766,660	308	£21.34		6%	12%			90%	596%		
	Zolpidem tartrate	631,844	12,767,623	£631,866	98,656,235	156	£1.00		11%	86%			75%	62%		
	Zopiclone	4,549,486	78,854,859	£4,651,478	473,873,599	104	£1.02		78%	86%			74%	79%		
	Total	5,821,236	107,582,996	£13,055,962	714,842,219	123	£2.24		100%	59%			60%	115%		

The overall reduction in dose per prescriptions may be partly explained through greater clinical focus on non‐pharmacological interventions such as talking therapies and social prescribing across all conditions. This is a particular issue in anxiolytics and hypnotics. Medicines wastage is also a key priority for the UK NHS currently, with prescribers potentially using shorter courses of psychotropics to control symptoms while other therapies are set‐up. Well publicised medicine addiction cases have also impacted on clinical behaviour with less emphasis on medicalising of psychological conditions. However, a significant number of patients still regard medicines as the most effective option to specifically address depression, which has contributed to the high growth in overall prescription volumes for antidepressants. It is important that patients and caregivers are educated about the rational use and careful use of these medications.

The phenomenon of price increase as volume of prescriptions falls has been reported in other pharmacotherapeutic areas (Heald et al. 2024). We did not include prescription of monoamine oxidase inhibitors moclobemide, tranylcypromine, and phenelzine due to very low volume of prescribing as previously reported (Heald et al. 2020). These prescriptions are usually for the indication of treatment‐resistant/atypical depression and in the United Kingdom, are made in collaboration with a specialist psychiatric clinic (Heald et al. 2020).

The observed increase in antidepressant medication prescribing can be interpreted in several different ways: it may be reflective of improved access to necessary medications, a reduction in the stigma surrounding mental health treatment, or more effective help‐seeking behaviour. On the other hand, it may reflect increased incidence or severity of psychiatric disorders or over‐prescribing. It is also possible that a combination of these factors is operating.

### 4.1 Strengths and Limitations

A strength of our analysis is that the data accounts for all English NHS primary care prescribing of antidepressants, anxiolytics and hypnotics for the period defined. However, time‐varying confounders such as the severity of depressive and anxiety disorders, socioeconomic status and health‐care access were not available.

We accept the limitation of the use of items prescribed as a measure of prescribing activity. The number of items in a prescription prescribed for a given item for a particular patient may vary; for example, 28‐day of medication could be prescribed as a single 28‐day prescription (one item) or four separate 7‐day prescriptions (four items). We were not able to differentiate prescribing by sex or age, as we used aggregated general practice level prescribing data year on year. Also the findings coming out of this study do only reflect prescribing patterns rather than the actual intake of these drugs by patients, as concordance was not assessed here.

We accept that antidepressants may be prescribed for other indications, such as neuropathic pain (particularly amitriptyline and duloxetine) and anxiety disorders (particularly SSRIs). Also the national prescribing datasets used do not contain information on indication. The majority of antidepressant prescribing is associated with a diagnosis of clinical depression.

## 5 Conclusions

In conclusion, the changes that we report here relate to changes in the guidance offered to primary care prescribers in relation to prescription of anxiolytics and hypnotics with increased access to alternative therapeutic approaches while the increase in antidepressant prescriptions may reflect broader societal factors.

Overall trends demonstrate a more than doubling of antidepressants prescribing over the period 2010–2023, with a similar volume reduction in anxiolytics and hypnotics over the same period. This increase in volume of antidepressant use was mirrored by a general downward trend in antidepressant prescribing costs of around 50%. Reasons for increased antidepressant use may be linked to reduced costs and changes in social attitudes to depression. The opposite trends in anxiolytics and hypnotic usage may be connected to greater awareness of the side effects of these medicines, with an accompanying increase in price, possibility as a market correction to lower demand.

Such real world data facilitate the development of policy influencing insights for psychotropic prescribing management now and in the coming years.

## Author Contributions

A.H. and M.S. conceived the study. M.D. provided essential input in relation to the evaluation of the findings as did D.T. Data analysis was undertaken by M.S. M.D., J.I. and J.W.L. provided context in relation to the implications of the findings. All authors reviewed the manuscript during its development, approved the final version and agree to be accountable for all aspects of the work.

## Ethics Statement

Ethical approval was not considered to be needed as we analysed publicly aggregated general practice level data. We did not access any individual patient data.

## Conflicts of Interest

The authors declare no conflicts of interest.

## Guarantor Statement

AH is the guarantor of this work and, as such, had full access to all the data in the study and takes responsibility for the integrity of the data and the accuracy of the data analysis.

## Supporting information

Supporting Information S1

## Data Availability

Data are available from the corresponding author on reasonable request.
