# Supplementary material for: National Prescribing Trends and Cost Analysis of Antidepressants, Anxiolytics, and Hypnotics in England, 2010–2023
Source: Hum Psychopharmacol. 2025 Aug 18;40(5):e70011. doi: 10.1002/hup.70011 (PMC12360015; doi:10.1002/hup.70011)
Supplement: Supplementary file 1 — Supporting Information S1 [file HUP-40-e70011-s001.docx]

#### Supplementary Table 1: BNF 0403 Prescribing analysis by Medication Top 17 Antidepressant taken as tablets/capsules in 2023 vs 2010

| **YEAR** | **Medication** | **TOTAL** | | | | **‘ / Precription (Px)** | | **Pxs % Total** | **Compare to 2010** | | | | |
| --- | --- | --- | --- | --- | --- | --- | --- | --- | --- | --- | --- | --- | --- |
|  |  | **Prescriptions** | **Quantity** | **Cost** | **Mg** | **Mg/** | **Cost/** |  | **Pxs** | | **mg/Px** | **£/Px** | |
| **2010** | Amitriptyline | 8,667,343 | 427,158,982 | £16,232,337 | 8,847,495,540 | 1,021 | £1.87 | 21% |  | |  |  | |
|  | Citalopram Hydrobromide | 11,823,077 | 383,983,564 | £20,243,125 | 7,776,357,320 | 658 | £1.71 | 28% |  | |  |  | |
|  | Clomipramine | 354,506 | 23,121,138 | £2,583,616 | 768,497,175 | 2,168 | £7.29 | 1% |  | |  |  | |
|  | Dosulepin | 1,707,029 | 87,086,573 | £4,234,724 | 4,044,369,675 | 2,369 | £2.48 | 4% |  | |  |  | |
|  | Duloxetine | 602,733 | 18,173,661 | £16,878,580 | 914,627,370 | 1,517 | £28.00 | 1% |  | |  |  | |
|  | Escitalopram | 1,285,853 | 40,526,205 | £24,815,492 | 498,813,780 | 388 | £19.30 | 3% |  | |  |  | |
|  | Fluoxetine | 5,283,089 | 223,693,348 | £19,791,607 | 4,559,976,580 | 863 | £3.75 | 13% |  | |  |  | |
|  | Flupentixol | 178,410 | 8,873,524 | £392,235 | 6,054,893 | 34 | £2.20 | 0% |  | |  |  | |
|  | Imipramine | 192,974 | 15,113,056 | £771,950 | 335,693,710 | 1,740 | £4.00 | 0% |  | |  |  | |
|  | Lofepramine | 339,024 | 19,322,143 | £2,897,265 | 1,352,550,010 | 3,990 | £8.55 | 1% |  | |  |  | |
|  | Mirtazapine | 3,361,238 | 85,185,492 | £11,894,508 | 2,418,123,564 | 719 | £3.54 | 8% |  | |  |  | |
|  | Nortriptyline | 323,866 | 21,268,461 | £3,417,017 | 319,543,605 | 987 | £10.55 | 1% |  | |  |  | |
|  | Paroxetine | 1,576,991 | 58,404,656 | £7,262,329 | 1,240,667,030 | 787 | £4.61 | 4% |  | |  |  | |
|  | Sertraline | 2,937,752 | 100,363,650 | £6,327,256 | 7,037,525,400 | 2,396 | £2.15 | 7% |  | |  |  | |
|  | Trazodone | 862,955 | 34,757,503 | £3,806,723 | 3,001,512,200 | 3,478 | £4.41 | 2% |  |  | | |  |
|  | Venlafaxine | 2,628,275 | 96,111,762 | £63,224,804 | 9,021,398,688 | 3,432 | £24.06 | 6% |  |  | | |  |
|  | **TOTAL** | **42,125,115** | **1,643,143,718** | **£204,773,565** | **52,143,206,540** | **1,238** | **£4.86** |  |  |  | | |  |
| **2023** | Amitriptyline | 15,623,774 | 774,992,287 | £20,585,548 | 12,608,531,470 | 807 | £1.32 | 18% | 180% | | 79% | 70% | |
|  | Citalopram Hydrobromide | 13,639,549 | 455,006,495 | £20,676,235 | 8,891,924,870 | 652 | £1.52 | 15% | 115% | | 99% | 89% | |
|  | Clomipramine | 225,269 | 13,017,486 | £3,155,147 | 477,114,170 | 2,118 | £14.01 | 0% | 64% | | 98% | **192%** | |
|  | Dosulepin | 343,424 | 18,141,611 | £4,571,279 | 790,580,875 | 2,302 | **£13.31** | 0% | 20% | | 97% | **537%** | |
|  | Duloxetine | 3,887,155 | 128,880,164 | £14,749,242 | 6,278,162,220 | 1,615 | £3.79 | 4% | **645%** | | 106% | 14% | |
|  | Escitalopram | 1,783,878 | 57,189,884 | £2,968,642 | 676,581,285 | 379 | £1.66 | 2% | 139% | | 98% | 9% | |
|  | Fluoxetine | 7,270,888 | 320,731,067 | £19,225,575 | 6,736,353,840 | 926 | £2.64 | 8% | 138% | | 107% | 71% | |
|  | Flupentixol | 126,364 | 5,664,754 | £367,182 | 4,275,856 | 34 | £2.91 | 0% | 71% | | 100% | 132% | |
|  | Imipramine | 107,880 | 8,430,872 | £444,420 | 176,620,250 | 1,637 | £4.12 | 0% | 56% | | 94% | 103% | |
|  | Lofepramine | 129,813 | 7,136,732 | £1,767,793 | 499,571,240 | 3,848 | £13.62 | 0% | 38% | | 96% | 159% | |
|  | Mirtazapine | 12,392,772 | 302,704,363 | £12,360,553 | 8,339,538,480 | 673 | £1.00 | 14% | **369%** | | 94% | 28% | |
|  | Nortriptyline | 875,223 | 55,333,793 | £3,115,236 | 803,977,005 | 919 | £3.56 | 1% | 270% | | 93% | 34% | |
|  | Paroxetine | 1,266,940 | 44,947,056 | £3,577,053 | 955,759,950 | 754 | £2.82 | 1% | 80% | | 96% | 61% | |
|  | Sertraline | 23,050,254 | 765,690,836 | £38,330,744 | 56,129,085,563 | 2,435 | £1.66 | 26% | **785%** | | 102% | 77% | |
|  | Trazodone | 1,226,272 | 42,291,423 | £2,979,962 | 3,762,414,100 | 3,068 | £2.43 | 1% | 142% | | 88% | 55% | |
|  | Venlafaxine | 5,728,750 | 203,069,942 | £41,193,750 | 20,348,736,338 | 3,552 | £7.19 | 7% | 218% | | 103% | 30% | |
|  | Vortioxetine | 339,017 | 8,271,424 | £8,188,710 | 97,134,910 | 287 | £24.15 | 0% |  | |  |  | |
|  | **TOTAL** | **88,017,222** | **3,211,500,189** | **£198,257,073** | **127,576,362,421** | **1,449** | **£2.25** |  | 209% | | 117%** | 46% | |

**% change averaged over medication weighted by the number of prescription (to avoid the effect of changes in mix)

#### Supplementary Table 2: BNF 0401 Prescribing analysis by Medication Anxiolytics and Hypnotics taken as tablets/capsules in 2023 vs 2010

| **YEAR** | **Medication** | **TOTAL** | | | | **‘ / prescription (Px)** | | | | **Pxs % Total** | | | **Compared to 2010** | | | | | | | |
| --- | --- | --- | --- | --- | --- | --- | --- | --- | --- | --- | --- | --- | --- | --- | --- | --- | --- | --- | --- | --- |
|  |  | **Prescriptions** | **Quantity** | **Cost** | **Mg** | **Mg** | **Cost/** | | |  |  |  | **Pxs** | **mg/Pxs** | | | | | **£/Px** | |
| **Anxiolytics** | | | | | | | | | | | | | | | | | | | | |
| **2010** | Buspirone | 146,842 | 7,995,304 | £3,866,333 | 51,624,345 | 352 | | £26.33 | 2% | | |  | | | |  | |  | | |
|  | Chlordiazepoxide | 213,761 | 9,915,081 | £894,219 | 72,683,000 | 340 | | £4.18 | 3% | | |  | | | |  | |  | | |
|  | Diazepam | 5,064,356 | 174,683,957 | £6,278,497 | 665,747,739 | 131 | | £1.24 | 77% | | |  | | | |  | |  | | |
|  | Lorazepam | 960,191 | 35,147,006 | £8,206,890 | 38,121,580 | 40 | | £8.55 | 15% | | |  | | | |  | |  | | |
|  | Oxazepam | 164,812 | 8,028,304 | £1,615,597 | 94,590,700 | 574 | | £9.80 | 3% | | |  | | | |  | |  | | |
|  | **TOTAL** | **6,549,962** | **235,769,652** | **£20,861,536** | **922,767,364** | **141** | | **£3.18** |  | | |  | | | |  | |  | | |
| **2023** | Buspirone | 217,868 | 11,616,181 | £2,304,989 | 75,464,838 | 346 | | £10.58 | 5% | | | 148% | | | | 99% | | 40% | | |
|  | Chlordiazepoxide | 26,165 | 1,016,398 | £307,220 | 7,085,750 | 271 | | **£11.74** | 1% | | | 12% | | | | 80% | | **281%** | | |
|  | Diazepam | 4,256,005 | 111,390,242 | £3,145,879 | 398,516,321 | 94 | | £0.74 | 89% | | | 84% | | | | 71% | | 60% | | |
|  | Lorazepam | 216,567 | 6,946,268 | £7,219,982 | 4,616,791 | 21 | | **£33.34** | 5% | | | 23% | | | | 54% | | **390%** | | |
|  | Oxazepam | 49,102 | 2,095,900 | £283,000 | 24,530,120 | 500 | | £5.76 | 1% | | | 30% | | | | 87% | | 59% | | |
|  | **TOTAL** | **4,765,707** | **133,064,989** | **£13,261,069** | **510,213,819** | **107** | | **£2.78** |  | | | 73% | | | | 76% | | 87% | | |
| **Hypnotics** | | | | | | | | | | | | | | | | | | | | |
| **2010** | Nitrazepam | 1,022,852 | 32,527,845 | £1,246,514 | 162,639,225 | 159 | £1.22 | | 10% | |  | | | |  | |  | | |  |
|  | Temazepam | 2,778,874 | 76,531,914 | £9,954,461 | 950,698,130 | 342 | £3.58 | | 28% | |  | | | |  | |  | | |  |
|  | Zolpidem Tartrate | 733,016 | 19,539,712 | £1,175,622 | 151,891,310 | 207 | £1.60 | | 7% | |  | | | |  | |  | | |  |
|  | Zopiclone | 5,289,867 | 125,202,630 | £6,858,848 | 748,584,619 | 142 | £1.30 | | 54% | |  | | | |  | |  | | |  |
|  | **TOTAL** | **9,824,609** | **253,802,101** | **£19,235,445** | **2,013,813,284** | **205** | **£1.96** | | 100% | |  | | | |  | |  | | |  |
| **2023** | Nitrazepam | 306,782 | 7,909,145 | £662,551 | 39,545,725 | 129 | £2.16 | | 5% | | 30% | | | | 81% | | 177% | | |  |
|  | Temazepam | 333,124 | 8,051,369 | £7,110,066 | 102,766,660 | 308 | **£21.34** | | 6% | | 12% | | | | 90% | | **596%** | | |  |
|  | Zolpidem Tartrate | 631,844 | 12,767,623 | £631,866 | 98,656,235 | 156 | £1.00 | | 11% | | 86% | | | | 75% | | 62% | | |  |
|  | Zopiclone | 4,549,486 | 78,854,859 | £4,651,478 | 473,873,599 | 104 | £1.02 | | 78% | | 86% | | | | 74% | | 79% | | |  |
|  | **TOTAL** | **5,821,236** | **107,582,996** | **£13,055,962** | **714,842,219** | **123** | **£2.24** | | 100% | | 59% | | | | 60% | | 115% | | |  |
